# A Retrospective Cohort Study Comparing the Outcomes of Conservative Versus Operative Fixation of Distal Radius Fractures in Children

**DOI:** 10.7759/cureus.22544

**Published:** 2022-02-23

**Authors:** Taibah H Aladraj, Ahmed S Keshta, Iftikhar Mukhtar, Anas A Zeidan, Mohammad A Abousaleh, Noora Ali

**Affiliations:** 1 Pediatric Orthopaedics, Royal College of Surgeons in Ireland - Bahrain, Busaiteen, BHR; 2 Pediatric Orthopaedics, Bahrain Defense Force (BDF) Hospital, Riffa, BHR; 3 Medicine, Royal College of Surgeons in Ireland - Bahrain, Busaiteen, BHR; 4 Pediatric Orthopaedics, Arabian Gulf University, Manama, BHR

**Keywords:** operative fixation, pediatrics, closed reduction, open reduction, distal radius fracture

## Abstract

Objectives

Distal radius fractures are common pediatric orthopedic injuries accounting for 25% of all fractures with a significant incidence in the age group 10-14 years. This study aims to evaluate the operative and non-operative methods of treating distal radius fractures in children.

Methods

This is a retrospective cohort study conducted on 176 children with distal radius fracture. We studied the operative and non-operative treatments of all children presented with distal radius fracture to the emergency department of the Bahrain Defense Force (BDF) Hospital from January 1, 2015, to February 1, 2022. The inclusion criteria were as follows: age of 17 years or younger, distal radius fracture with or without complete displacement and skeletal immaturity managed as of non-operative or operative groups. Patients who did not have follow-up data after the date of surgery were excluded. The statistical analysis was performed using the software SPSS version 23.0 (IBM Corp., Armonk, NY). Continuous data expressed as mean, standard deviation and discrete variables were expressed as frequency and percentages. One-way Analysis of Variance (ANOVA) was used to compare the continuous variables between groups. The Student’s t-test was used for the two-group comparison. For the comparison of discrete variables, a Chi-Square test or Fisher’s exact test was used.

Results

Seventy-seven patients were conservatively managed with cast immobilization (“non-operative” group) in comparison to 99 patients who were surgically managed (“operative” group) with either percutaneous pinning (n=56) or flexinail (n=43). Fewer patients underwent physiotherapy in the operative group with 14 (25.0%) patients for percutaneous pinning and seven (16.3%) patients for flexinail versus 31 (40.3%) patients in the non-operative group (p<0.015). There were statistically significant differences in radial inclination (p<0.001) between conservative and percutaneous pinning (22.22±2.86 vs 18.76±3.33 degrees) and percutaneous pinning and flexinail (18.76±3.33 vs 22.37±3.44 degrees). Likewise, there was a significant difference found in ulnar variance between conservative and percutaneous pinning (-0.45±2.14 mm vs -1.47±1.93 mm, p=0.012) and conservative and flexinail (-0.45±2.14 mm vs -1.59±1.90 mm, p=0.009). There were a total of 25 documented complications. Nineteen (19.8%) complications occurred in the non-operative group versus five (7.2%) and one (2.3%) complications in percutaneous pinning and flexinail groups, respectively (p=0.003). The most common complication in the non-operative group was loss of reduction while in cast and subsequent need for surgical intervention. Ten of these patients underwent percutaneous pinning whereas nine were fixed by flexinail.

Conclusion

This study illustrated an overall similar success between the surgical and the conservative treatments of distal radius fractures in children. Due to the higher complication rate reported in the conservative group, the conservative treatment cannot be considered safer than the surgical treatment.

## Introduction

Distal radius fractures (DRFs) are common orthopedic injuries accompanied by suffering and substantial health care costs [1-2]. Statistics show a significant increase in the incidence of DRFs in children and adolescents [3]. In the pediatric population, DRFs represent 25% of all fractures with a significant incidence in the age group 10-14 years [4]. The subsequent loss of reduction occurs in 5% to 75% of pediatric forearm fractures [5]. The closed reduction of pediatric forearm fractures is implemented with a known physiological range of supination and pronation motion. Frequently, with clinical and radiographic follow-up, there will be no additional interventions after the initial closed reduction which results in the reduction of costs and radiation exposure [6]. Consequently, most conservative managements restore normal function to the limb with the cast application [2]. Cast is applied under conscious sedation which can be either short arm casting (SAC) or long arm casting (LAC). Cast index is a well-grounded quality marker with a desired approximate ratio of up to 0.7 sagittal/coronal widths [7]. Operative intervention is recommended when an acceptable reduction cannot be maintained. In other words, surgeries are indicated when unstable reduction or loss of reduction in cast occurs with respect to the degree of angulation and rotation of the limb [6]. The current options of surgical interventions include closed reduction with percutaneous pinning (k-wire) or closed/open reduction with internal fixation [7]. Intramedullary nails are commonly considered for internal fixation of pediatric forearm fractures due to their safety, short anesthetic duration, short hospital stay, and easy removal following placement [6]. Finally, several complications should be kept in the surgeon’s mind such as neuropathy, stiffness, tendonitis, malunion, arthrosis, carpal tunnel syndrome, compartment syndrome, and infection [8].

## Materials and methods

This is a retrospective cohort study conducted on children with distal radius fracture. We studied the operative and non-operative treatment of children who presented with distal radius fracture to the emergency department of the Bahrain Defense Force (BDF) Hospital from January 1, 2015, to February 1, 2022. The inclusion criteria were as follows: age of 17 years or younger, distal radius fracture with or without complete displacement, and skeletal immaturity managed as of non-operative or operative groups. Patients who did not have follow-up data after the date of surgery were excluded. The functional and radiological outcomes of both management methods were compared. In order to have a fixed medical background of both groups, characteristics of patients including age, gender, mechanism of injury, laterality fractured bone and neurovascular status were studied.

The functional outcomes were mainly evaluated by a range of motion (ROM), grip strength, and Gartland and Werley grade. ROM containing flexion, extension, supination, and pronation was rated as: full motion, restricted motion, or no motion. Grip strength was rated as: full strength, reduced strength, or no strength. Both outcomes were assessed by clinical examination based on comparison to the contralateral uninjured wrist. Gartland and Werley scoring system is composed of four categories: residual deformity, pain, ROM, and complications. The higher the score the poorer the outcome that a score of 0-2 is excellent, 3-8 is good, 9-20 is fair and 21 or more is poor [9].

Radiological outcomes including radial inclination, radial height, volar tilt, ulnar variance, and articular step-off were assessed using antero-posterior and lateral X-rays. Articular step-off of 2 mm or greater was defined as significant [10]. Patients were evaluated for complications including loss of reduction, infection, tendon tenosynovitis, compartment syndrome, delayed or non-union, neuropathy. Delayed union was defined as no evidence of trabecular bridging at four months while non-union was defined as no evidence of trabecular bridging at six months.

All children-related data analysis was statistically performed using the software SPSS version 23.0 (IBM Corp., Armonk, NY). Nonparametric analyses were used, as the outcomes are based on relatively small sample size. Continuous data expressed as mean and standard deviation (SD) and discrete variables were expressed as frequency and percentages. One-way Analysis of Variance (ANOVA) was used to compare the continuous variables between groups. The Student’s t-test was used for the two-group comparison. For the comparison of discrete variables, a Chi-Square test or Fisher’s exact test was used. The P-value was set at 0.05 (95% CI) to indicate statistically significant results. This study was approved by the Research Ethics Committee of the Royal Medical Services - Bahrain Defense Force (RMS-BDF) on 14th March 2021.

## Results

A total of 176 patients were included. Seventy-seven patients were conservatively managed with cast immobilization (“non-operative” group) in comparison to 99 patients who were surgically managed (“operative” group) with either percutaneous pinning (n=56) or flexinail (n=43). Table 1 illustrates the demographic differences between the groups being studied.

**Table 1:** Demographic characteristics of children in the non-operative versus the operative group (Percutaneous pinning and Flexinail) p<0.05 considered as statistically significant. *Comparison between three groups, p-value from one-way ANOVA. **p-value from Chi-square test or Fisher’s exact test.

		Surgical techniques (n=99)		
Variables	Non-Operative (n=77)	Percutaneous pinning (n=56)	Flexinail (n=43)	P-value
Age at injury (years) (mean±SD)	7.61±3.12	9.52±3.50	8.47±4.08	0.009*
Gender				
Male	67 (87.0%)	48 (85.7%)	33 (76.7%)	0.315
Female	10 (13.0%)	8 (14.3%)	10 (23.3%)	
Laterality				
Left	38 (49.4%)	31 (55.4%)	19 (44.2%)	0.558
Right	39 (50.6%)	25 (44.6%)	24 (55.8%)	
Mechanism of injury				
Fall	77 (100.0%)	55 (98.2%)	41 (95.3%)	0.101
Road traffic accident (RTA)	0 (0.0%)	1 (1.8%)	2 (4.7%)	
Fractured Bone				
Radius alone	59 (76.6%)	44 (78.6%)	24 (55.8%)	0.022*
Radium and ulna	18 (23.4%)	12 (21.4%)	19 (44.2%)	
Neurovascular				
Intact	77 (100.0%)	54 (96.4%)	43 (100.0%)	0.159
Partial	0	2 (3.6%)	0	

The median follow-up time was longer for the operative group with 3.66±3.53 months for percutaneous pinning and 7.32±2.58 months for flexinail versus 1.65±1.00 for the non-operative group (p<0.001). The time of metal removal between percutaneous pinning compared to flexinail was significantly different (p<0.001) with 1.33±1.13 months and 6.47±2.57 months, respectively. However, there was no significant difference in the immobilization time between groups. Fewer patients underwent physiotherapy in the operative group with 14 (25.0%) patients for percutaneous pinning and seven (16.3%) patients for flexinail versus 31 (40.3%) patients in the non-operative group (p<0.015) (Table 2).

**Table 2:** A comparison of follow-up characteristics of children in the non-operative versus the operative group (Percutaneous pinning and Flexinail) p<0.05 considered as statistically significant. *Comparison between three groups, p-value from one-way ANOVA. **p-value from Chi-square test or Fisher’s exact test.

		Surgical techniques (n=99)		
Variables	Non-Operative (n=77)	Percutaneous pinning (n=56)	Flexinail (n=43)	P-value
Follow-up period (months) (mean±SD)	1.65±1.00	3.66±3.53	7.32±2.58	<0.001*
Time for metal removal (months) (mean±SD)	NA	1.33±1.13	6.47±2.57	<0.001*
Immobilization time (months) (mean±SD)	1.28±0.21	1.59±1.50	1.22±0.51	0.069
Physiotherapy				
Done	31 (40.3%)	14 (25.0%)	7 (16.3%)	0.015*
Not done	46 (59.7%)	42 (75.0%)	36 (83.7%)	

Clinical assessment of ROM and grip strength showed no significant difference between groups. No patient experienced the loss of wrist motion, whereas one (1.8%) patient in the percutaneous pinning group experienced the loss of grip strength. Similarly, outcomes of Gartland and Werley scale at the last visit showed no significant difference between groups. Most patients scored excellent, and the remaining patients scored good. Only one (1.8%) patient in the percutaneous pinning group scored fair (Table 3).

**Table 3:** Functional outcomes of children in the non-operative versus the operative group (Percutaneous pinning and Flexinail)

			Surgical techniques (n=99)		
	Variables	Non-Operative (n=77)	Percutaneous pinning (n=56)	Flexinail (n=43)	P-value
Range of motion (Flexion)	Full motion	74 (96.1%)	54 (96.4%)	42 (97.7%)	>0.05
	Restricted motion	3 (3.9%)	2 (3.6%)	1 (2.3%)	
	No motion	0	0	0	
Range of motion (Extension)	Full motion	77 (100.0%)	55 (98.2%)	42 (97.7%)	0.315
	Restricted motion	0	1 (1.8%)	1 (2.3%)	
	No motion	0	0	0	
Range of motion (Pronation)	Full motion	77 (100.0%)	55 (98.2%)	43 (100.0%)	0.563
	Restricted motion	0	1 (1.8%)	0	
	No motion	0	0	0	
Range of motion (Supination)	Full motion	77 (100.0%)	55 (98.2%)	43 (100.0%)	0.563
	Restricted motion	0	1 (1.8%)	0	
	No motion	0	0	0	
Grip Strength	Full Strength	77 (100.0%)	54 (96.4%)	43 (100.0%)	0.315
	Reduced strength	0	1 (1.8%)	0	
	No strength	0	1 (1.8%)	0	
Gartland & Werley grade	Excellent	67 (87.0%)	44 (78.6%)	35 (81.4%)	0.487
	Good	10 (13.0%)	11 (19.6%)	8 (18.6%)	
	Fair	0	1 (1.8%)	0	
	Poor	0	0	0	

There were statistically significant differences in radial inclination (p<0.001) between conservative and percutaneous pinning (22.22±2.86 degrees vs 18.76±3.33 degrees) and percutaneous pinning and flexinail (18.76±3.33 degrees vs 22.37±3.44 degrees). Likewise, there was a significant difference found in ulnar variance between conservative and percutaneous pinning (-0.45±2.14 mm vs -1.47±1.93 mm, p=0.012) and conservative and flexinail (-0.45±2.14 mm vs -1.59±1.90 mm, p=0.009). There was no significant difference in radial height or palmar tilt between groups. There was no case of significant articular step-off (Table 4).

**Table 4:** Radiological findings of children in the non-operative versus the operative group (Percutaneous pinning and Flexinail) *Statistical significance between three groups (One-Way ANOVA). †Statistically significant difference between percutaneous vs flexinail, percutaneous vs non-operative

		Surgical techniques (n=99)		
Variables	Non-Operative (n=77)	Percutaneous pinning (n=56)	Flexinail (n=43)	P-value
Radial inclination (degrees) (mean±SD)	22.22±2.86	18.76±3.33†	22.37±3.44	<0.001*
Radial Height (millimeters) (mean±SD)	8.62±2.34	8.60±2.04	9.29±3.18	0.306
Volar tilt (degrees) (mean±SD)	9.56±4.37	9.64±4.85	8.89±3.79	0.655
Ulnar variance (millimeters) (mean±SD)	-0.45±2.14‡	-1.47±1.93†	-1.59±1.90	0.002*
Articular step off (millimeters)	-	-	-	-

There were a total of 25 documented complications. Nineteen (24.7) complications occurred in the non-operative group versus four (7.2%) and one (2.3%) complications in percutaneous pinning and flexinail groups, respectively (p=0.003). The most common complication in the non-operative group was loss of reduction while in cast and subsequent need for surgical intervention, 10 of these patients underwent percutaneous pinning whereas nine were fixed by flexinail. The complications in the pinning group included pin site infection (2, 3.6%), physeal growth arrest (1, 1.8%), and neuropathy (1, 1.8%). There was one case of infection in the flexinail group (1, 2.3%).

## Discussion

To date, the results of most DRFs management studies are non-conclusive, with no difference between conservative and surgical management and outweighing a method over the other has been established [8, 11]. Likewise, we present a relatively similar overall outcome between the operative and the non-operative groups. Upon evaluating the characteristics of both groups, the mean age at which the injury occurred was approximately eight years, which is less than Ozcan et al.’s findings with approximately average age of 10 years [8]. Although the follow-up period for the operative group was longer than the non-operative group, the need for physiotherapy in the non-operative group was remarkably higher. This should be further studied as it might indicate a relative superiority in the success of surgical management over the conservative management.

The functional assessment of the patients including, range of motion, grip strength, and Gartland and Werley grade was not of any impact in outweighing one method over the other. This has been demonstrated in previous studies including 68 and 40 patients with similar results of significance [8, 11]. There was a significant difference in the rate of complications occurring more in the non-operative than the operative groups. In addition, the nature of complications was distinct between the two groups. The most common complication in the non-operative group was the loss of reduction with a rate of 24.7%. This rate lies within the range reported in the available literature, which is between 21% to 39% [12]. The re-displacement of distal radius fracture in children can be a result of several factors including, initial complete displacement, degree of obliquity of the fracture, and an association of ulnar fracture when distal radius fracture is managed conservatively. Therefore, percutaneous pinning is suggested for children with a high risk of re-displacement of distal radius fractures who are managed conservatively [13,14]. On the other hand, the surgical intervention was found to be commonly associated with superficial pin-site infection. All three patients were managed by cast change and antibiotics, none required pin removal. There was one case of ulnar growth arrest in a 13-year-old boy who was managed by k wire; this was diagnosed via X-ray done one year post operation with an ulnar variance of -7.8 mm. The patient had full range of motion and mild point tenderness in the ulnar side, but no intervention was indicated.

It is worth noting that the amount of literature available on surgical treatment is much larger than that on conservative treatment of DRFs. In fact, a PubMed search using the terms “surgical treatment of distal radius fracture” provides over 4,000 results. In contrast, a search using the terms “conservative treatment of distal radius fracture” provides a list of 300 results for almost the same period of time. Therefore, a comparative discussion using the available literature is prone to selection and/or reporting bias. Additionally, the loss of follow-up and the absence of the long-term follow-up (5-10 years) in the study, had an essential role in the modifying evaluation of the outcomes of both groups. Finally, the relatively small sample size and the retrospective nature were major limitations of this study that might have had a negative impact on the accuracy of the study findings.

## Conclusions

To conclude, this study illustrated an overall similar success between the surgical and the conservative treatments of distal radius fractures in children. Unlike the widely known superiority of conservative over surgical treatment in terms of patient safety, this study presents a higher complication rate of the conservative management than the surgical management. Therefore, the conservative treatment cannot be considered a safer option than the surgical treatment. Finally, a larger dedicated international effort has been directed towards studying the surgical intervention alone. This highlights the need for a cautious approach when drawing parallels between surgical and conservative managements, despite the obvious and significant similarities between both management methods.
